# Structural insights into DDA1 function as a core component of the CRL4-DDB1 ubiquitin ligase

**DOI:** 10.1038/s41421-018-0064-8

**Published:** 2018-12-11

**Authors:** Nitzan Shabek, James Ruble, Claire J. Waston, Kenneth C. Garbutt, Thomas R. Hinds, Ti Li, Ning Zheng

**Affiliations:** 1 Department of Pharmacology, University of Washington, Box 357280, Seattle, WA 98195 USA; 2 Howard Hughes Medical Institute, University of Washington, Box 357280, Seattle, WA 98195 USA; 3 Present Address: Department of Plant Biology, University of California - Davis, Davis, CA 95616 USA

Dear Editor,

Cullin-RING ligases (CRLs) represent the largest super-family of multi-subunit ubiquitin E3 ligases and regulate a wide range of cellular functions in all eukaryotes^1^. As a structurally distinct sub-family of CRLs, the CRL4 E3 complexes consists of three well-defined basal subunits—a cullin4 (CUL4A or CUL4B) scaffold protein, a RING finger domain catalytic subunit, RBX1, and a large adaptor subunit, DDB1 (DNA damage-binding protein 1). With a unique triple β-propeller (BPA, BPB, and BPC) topology, DDB1 recruits and coordinates a large variety of substrate receptors, known as DCAFs (DDB1 and Cullin4-Associated Factors), which in turn recognize specific substrate proteins for ubiquitination^2–4^ (Fig. 1a). CRL4s play an important role in many essential cellular processes, such as transcription, cell cycle progression, DNA damage repair, and chromatin remodeling, and are frequently hijacked by pathogenic viruses^5^. A number of substrates have been identified for CRL4s, which include p21, Cdt1, DDB2, XPC, and histones^4^. Recent studies have further uncovered several neo-substrates of CRL4, which are recruited to DCAF proteins, such as CRBN and DCAF15, by therapeutic and investigational compounds^6–9^. Major advances in our understanding of CRL4 function have benefited greatly from early structural studies, which started by revealing the unique architecture of its central component, DDB1^2,5^. Distinct from the adaptor subunits of other CRLs, DDB1 uses one of its β-propeller domains, BPB, to anchor at the N-terminal region of CUL4A. Its two other β-propeller domains, BPA and BPC, form an interwoven double-propeller fold that is shaped like a half-open clamshell, and provide a binding surface for DCAF substrate receptors (Fig. 1a). Interestingly, the DDB1 triple propeller has a restrained intrinsic flexibility, which allows a wide range of spatial configuration between the BPB domain and the BPA-BPC double-propeller^2^.

**Fig. 1:**
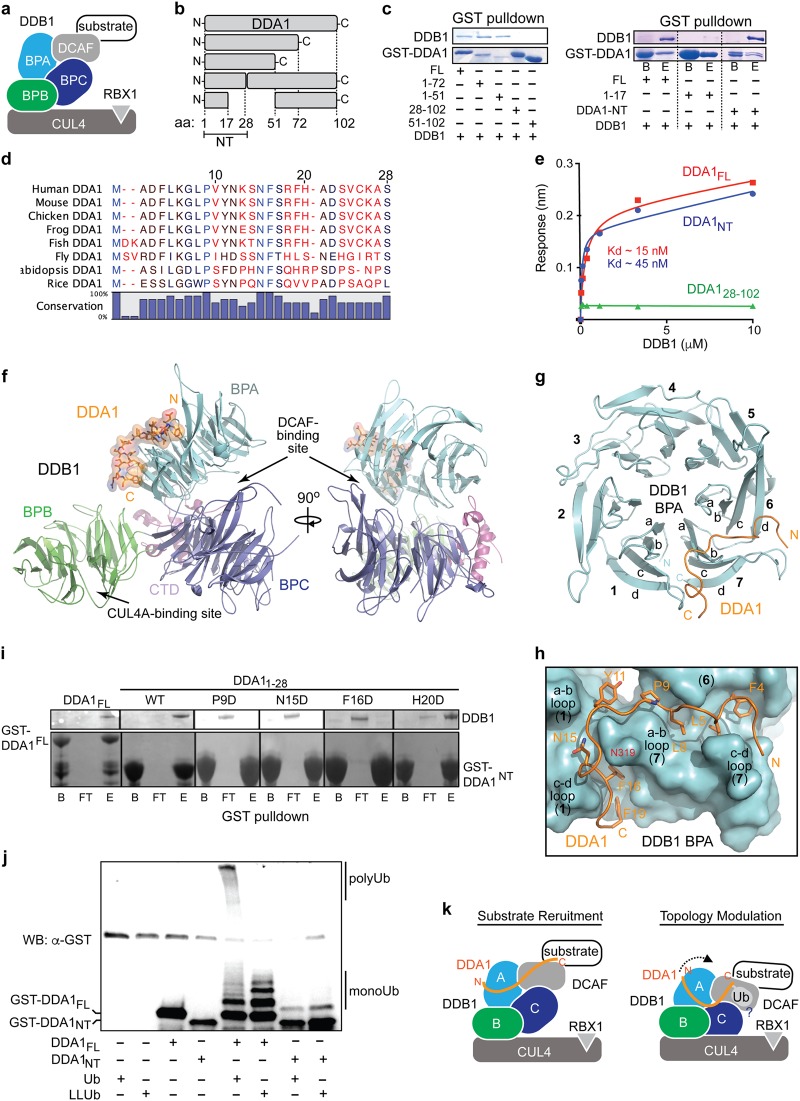
Structural characterization of DDA1–DDB1 complex. **a** Schematic representation of CRL4 ubiquitin ligase complex. The three DDB1 domains are colored and labeled BPA, BPB, and BPC. **b** DDA1 truncation constructs used to test interactions with DDB1. **c** GST pull-down assays showing direct interactions between DDB1 and DDA1 with a minimal requirement of 1–28 aa N-terminal region (NT). B beads with GST-fused bait, E elution, FL full length. **d** Sequence alignment and conservation of DDA1-NT across animal and plant species. **e** Bio-layer interferometry analysis of DDB1 binding by full-length (FL) DDA1, DDA1-NT (1–28 aa), and DDA1-CT (28–102 aa). Affinity-tagged DDA1 FL and fragments were immobilized on the probe as ligand. Free DDB1 was included as analyte at different concentration. Kd is calculated based on the dose response curve made with maximal binding signals. **f** Orthogonal views of the DDB1–DDA1 complex. DDA1 is shown in sticks (orange) and semi-transparent surface representation. The BPA, BPB, BPC, and CTD domain of DDB1 are shown in cyan, green, slate, and magenta cartoon diagrams. **g**–**h** Overall and close-up views of the interface between DDA1 (orange) and DDB1 BPA domain (cyan). DDA1 residues involved in DDB1 binding are shown in orange sticks. The seven blades of DDB1-BPA are labeled “1”–“7”. The four b-strands of blade “1”, “6”, and “7” are labeled “a” to “d”. **i** GST pull-down assay testing the binding of purified DDB1 to glutathione-beads-immobilized GST-tagged DDA1 FL, NT, and NT mutants as indicated. B, FT, and E stand for beads with GST-fusion bait, flow-through, and eluates, respectively. **j** In vitro reconstituted ubiquitination of GST-DDA1 (FL or NT) in the presence of wild type or lysine-less ubiquitin (denoted Ub and LLUb, respectively). **k** Two models illustrating the putative mode of action of DDA1 in CRL4 ligase function. DDA1 is anchored at the back side of the DDB1 BPA domain and might use its flexible C-terminal region to recruit a DCAF–substrate complex (left) and/or modulate the overall architecture of the substrate-loaded CRL4 E3 complex (right). A question mark and a ubiquitin molecule (Ub) indicate the possibility that DDA1 ubiquitination might be involved in its mode of action

DDA1 (DDB1 and DET1 associated 1) has emerged as a fourth evolutionarily conserved basal component of the CRL4 core complex^10^. DDA1 was first identified as a subunit of the plant DDD (DDB1–DET1–DDA1) complex, which binds COP10 and together plays a role in repressing photomorphogenesis^11,12^. Its animal ortholog was also found in mammalian CRL4s and the DDD-E2 complex (DDB1–DET1–DDA1–UbE2E-E2)^2,12^. While it is well established that aberrant expression of CUL4 is observed in multiple tumors, an increasing number of reports have also suggested a significant contribution of DDA1 as an oncogene^13^. Interestingly, DDA1 has been recently documented to participate in plant hormone abscisic acid signaling and reprogramming of CRL4s by therapeutic compounds for neo-substrate ubiquitination and degradation^7–9,14^. Despite this increasing body of evidence implicating DDA1 in CRL4 function and regulation, how it interacts with the other CRL4 subunits and potentially contributes to CRL4 E3 activity remained largely unknown.

To better understand the role of DDA1 in CRL4, we first confirmed the direct interaction between DDA1 and DDB1 with purified recombinant proteins and subsequently mapped the region of DDA1 responsible for binding DDB1 (Fig. 1b). Consistent with previous studies^10^, we found that the highly conserved N-terminal 28 amino acids sequence of DDA1 (DDA1-NT) is necessary and sufficient for engaging DDB1 (Fig. 1c, d, Supplementary Fig. S1a). Using bio-layer interferometry, we further quantified the binding between DDA1 and DDB1, which revealed a high DDB1-binding affinity of DDA1-NT, similar to the full-length protein (Kd ~45 nM, Fig. 1e). In these experiments, the DDA1-CT fragment complementary to DDA1-NT showed no detectable interaction with DDB1 (Fig. 1c).

In order to map the DDA1-binding site on DDB1, we crystallized and determined the structure of DDB1 in complex with DDA1-NT at a resolution of 3.1 Å (Fig. 1f, Supplementary Table S1). The complex structure revealed clear electron density for the N-terminal 19 amino acids region of DDA1 (amino acids 4–19), which adopts a partially coiled conformation. Remarkably, the DDA1-NT fragment is docked to the bottom surface of the DDB1 BPA domain, which is far removed from both the CUL4A- and DCAF-binding sites (Fig. 1f). Similar to many β-propeller folds, the DDB1 BPA domain contains seven four-stranded blades. From the inner to outer position, the four β-strands constructing each blade are conventionally named from “a” to “d” (Fig. 1g). The bottom surface of a β-propeller fold is decorated by loops connecting strand a to b and c to d. The DDA1-NT fragment anchors to a continuous hydrophobic groove that is formed between blade 6 and 7 and blade 7 and 1 (Fig. 1g). This DDA1-binding groove of DDB1 is marked by residues that are highly conserved among metazoans (Supplementary Fig. S1b). Upon binding to this groove, DDA1-NT creates a partition between blade 7 and the remaining portion of the BPA domain. Most DDA1-NT contacts are mediated by a–b and c–d loops of blade 1 and 7 in the BPA domain and the unusually long c- and d-strands of blade 6 (Fig. 1g). In comparison to the free DDB1 structure, DDA1-NT binding does not significantly alter the DDB1 BPA structure (Supplementary Fig. S1c).

DDA1-NT contains several conserved hydrophobic residues, which occupy the surface cavities along the BPA groove (Fig. 1h and Supplementary Fig. S1d). The ~20 amino acids DDA1 fragment can be separated at Pro9 into two equal halves. The N-terminal half of DDA1-NT is sandwiched between blade 6 and 7 of the DDB1 BPB domain, where three DDA1 residues, Phe4, Leu5, and Leu8, secure the inter-molecular binding through hydrophobic interactions (Fig. 1h). The C-terminal half of DDA1-NT, meanwhile, meanders along the junction between blade 1 and 7, making both polar and hydrophobic interacts with DDB1 via Asn15, Phe16, and Phe19 (Fig. 1h). Noticeably, Pro9, Asn15, and Phe16 are strictly conserved between animals and plants (Fig. 1d), underscoring the importance of the C-terminal half of the DDA1-NT fragment for DDB1 association. To validate our structure and the importance of the DDA1 residues at the interface, we made and purified the DDA1-NT fragment with single amino acid mutations and assessed their DDB1-binding activities through a GST pull-down assay. In contrast to His20, which is disordered in the crystal structure, replacement of Pro9, Asn15, or Phe16 with a negatively charged residue was sufficient to abolish DDB1–DDA1-NT complex formation (Fig. 1i). Removal of Arg18 and Phe19 also abrogated the interaction (Fig. 1c). Interestingly, the tomato *hp-1* mutant, which is characterized by an exaggerated photoresponsiveness phenotype, has been previously reported to harbor a single missense mutation at Asn311 of tomato DDB1^15^. The corresponding residue in human DDB1, Asn319, directly participates in DDA1 binding by donating two hydrogen bonds to the backbone carbonyls of DDA1-NT (Fig. 1h). The critical role of this portion of the DDB1–DDA1 interface is further corroborated by the previously documented DDB1 triple mutant, Y316/D318/N319, which loses its ability to bind DDA1^3^.

Our binding and structural analyses establish the binding mode of DDA1 on DDB1, which involves a close interaction mainly between the extreme N-terminal 20 amino acids region of DDA1 with the backside of DDB1 double-propeller fold. This finding raises an immediate question about the structural property and functional role of DDA1 C-terminal region within the CRL4 E3. Although this portion of DDA1 contains several conserved hydrophobic residues (Supplementary Fig. S1a), it is unlikely to adopt a globular fold on its own. We hypothesized that, upon binding to DDB1, the majority of the DDA1 C-terminal region is able to sample a large open space surrounding the CUL4 adaptor. To probe its spatial relationship to the rest of the E3 complex, we took advantage of the CRL4′s E3 activity and performed an in vitro ubiquitination assay with purified CUL4A–RBX1–DDB1 in complex with GST-fused DDA1-NT and DDA1 full-length protein (Fig. 1j). Consistent with the far distance between its docking site on DDB1 and RBX1, ubiquitin modification of GST-DDA1-NT is inefficient and mostly limited to mono-ubiquitin conjugation. By contrast, the GST-fused full-length DDA1 protein was both mono- and poly-ubiquitinated, indicating that the DDA1 C-terminal region rendered the fusion protein more accessible to RBX1-bound E2. Although the physiological significance of DDA1 ubiquitination remains to be investigated, we speculate that the C-terminal region of DDA1 can reach DCAF or even DCAF-bound substrate on the other side of the DDB1 double-propeller fold to either facilitate substrate recruitment or modulate the overall topology of the fully assembled CRL4–substrate complex (Fig. 1k).

## Electronic supplementary material

Supplementary Information

## Electronic supplementary material

**Supplementary Information** accompanies the paper at 10.1038/s41421-018-0064-8.
