# Supplementary material for: Structural insights into DDA1 function as a core component of the CRL4-DDB1 ubiquitin ligase
Source: Cell Discov. 2018 Dec 11;4:67. doi: 10.1038/s41421-018-0064-8 (PMC6288126; doi:10.1038/s41421-018-0064-8)
Supplement: Supplementary file 1 — Supplementary Information [file 41421_2018_64_MOESM1_ESM.pdf]

Structural Insights into DDA1 Function as A Core Component of the CRL4-DDB1 Ubiquitin Ligase

Nitzan Shabek[1,2,*,‡], James Ruble[1*], Claire J. Waston[1], Kenneth C. Garbutt[1], Thomas R. Hinds[1], Ti Li[1], & Ning Zheng[1,2,**]

[1]*Department of Pharmacology and* [2]*Howard Hughes Medical Institute, Box 357280, University of Washington, Seattle, WA 98195.*

[‡]*Current Address: Department of Plant Biology, University of California – Davis, Davis, CA 95616.*

**Equal contribution*

***Correspondence should be addressed to: nzheng@uw.edu*

Supplementary Information

1. DDB1-DDA1 sequence and structure analysis: **Supplementary Figure S1**
2. Detailed experimental procedures: **Methods**
3. Data collection and refinement statistics: **Supplementary Table S1**

Supplementary Figure S1

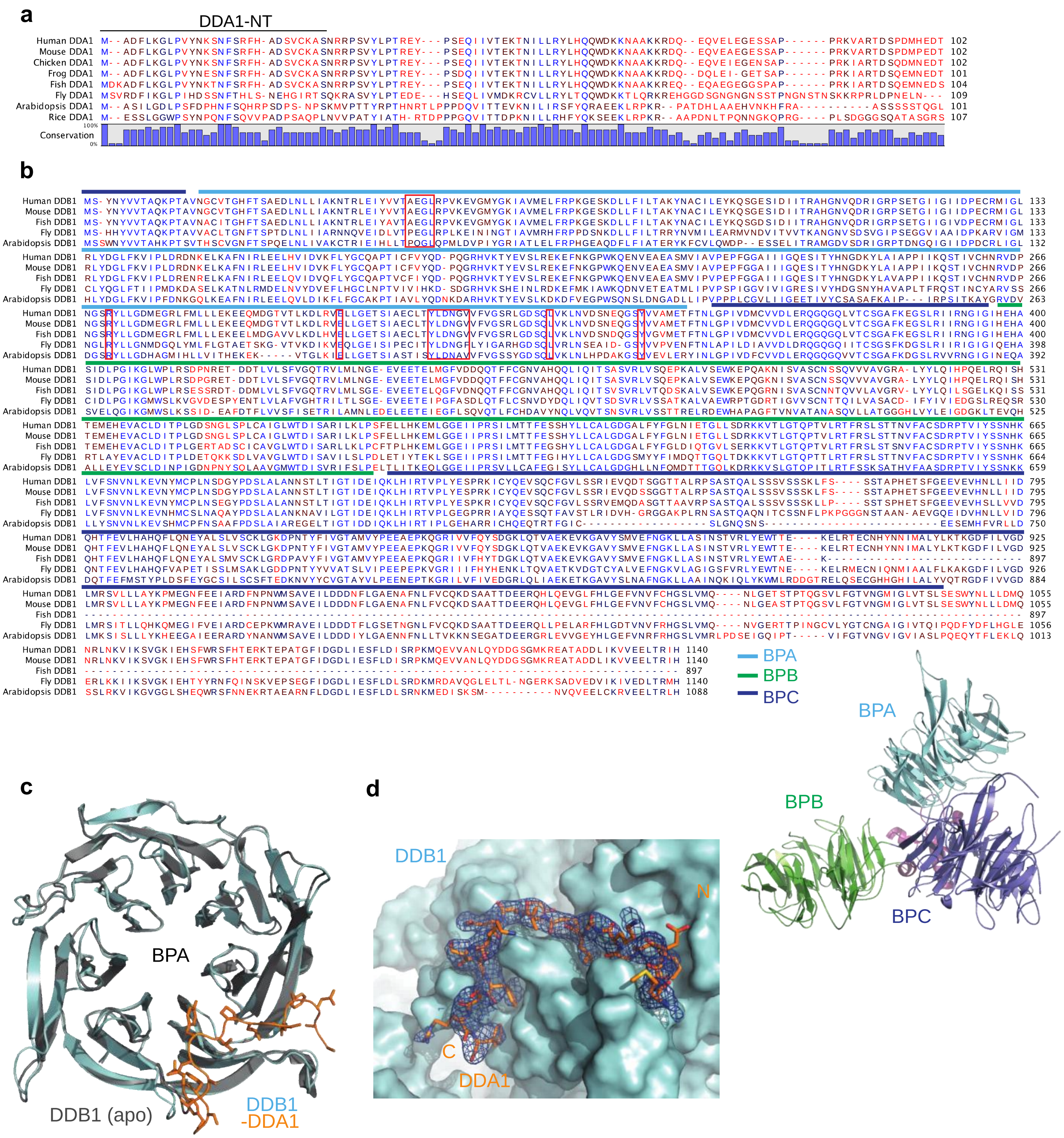

Supplementary Figure S1. (a) Sequence alignment and conservation of DDA1 across representative species. The N-terminal 28 residues of DDA1 is indicated as DDA1-NT. **(b)** Alignments of DDB1 sequences across representative species. The three BP domains are highlighted by solid lines with the same color scheme used in the DDB1 overall structure shown underneath on the right (BPA: cyan, BPB: green, and BPC: slate). Residues involved in binding DDA1 are highlighted by red frame. **(c)** Superposition of the BPA domain of DDB1 in its apo form (grey, PDB 2B5M) and DDA1-complexed form (cyan). DDA1 is shown in orange cartoon. **(d)** Composite omit map (blue) contoured at 1.0σ showing the density of DDA1 (orange in stick representation) in complex with DDB1 (cyan in surface representation). Most of the DDB1 facing residues in DDA1 have well-defined density.

Methods

Protein expression and purification

Human DDB1 was overexpressed with an N-terminal 6xHis tag in Hi5 insect cells. Protein purification from cell lysate was performed with a nickel column and affinity chromatography as described previously[1]. DDA1, DDA1-NT, and all other DDA1 truncations and point mutants were expressed in *E. coli* with N-terminal GST tags, and initial purification was performed with a glutathione agarose column. All protein tags contained TEV protease cut sites which were used for removal of the GST tag. Further purification was performed with anion exchange and gel filtration columns. Final protein buffers consisted of 20 mM Tris pH 8.0, 200 mM NaCl, and 5 mM DTT. Synthesized DDA1-NT peptide used for crystallography was obtained from Bio-Synthesis and mixed with DDB1 at 1:1.5 ratio.

Crystallization, data collection and structure determination

The crystals of DDB1-DDA1 complex were grown at 4 ℃ by the hanging-drop vapor diffusion method with 1.0 μL protein complex sample mixed with an equal volume of reservoir solution containing 0.1 M MES monohydrate, pH 6.5, 20% (w/v) PEG4000, 180 mM NaCl, 5 mM DTT. X-ray diffraction data were integrated and scaled with HKL2000 package[2]. The DDB1-DDA1 structure was determined by molecular replacement using DDB1 structure (PDB: 2B5M)[3] as the search model. Structural model was manually built, refined, and rebuilt with PHENIX[4] and COOT[5]. The DDB1-DDA1-NT crystal suffered from radiation damage and prevented a complete dataset from being collected at 3.1 Å resolution. A poly-alanine DDA1 model was first built and refined before the side chains were assigned with the help of NCS density averaging.

Binding assays

Pull-down assay was preformed using ~10-50 μg of purified GST-tagged proteins as the bait and ~ 15-30 μg of no-tagged DDB1. Reaction mixtures were incubated with GST beads (GE Healthcare) at 4 °C for 30 minutes in a reaction buffer containing 50 mM Tris-HCl, pH 7, 100 mM NaCl 2 mM DTT. After extensive wash with a buffer containing 50 mM Tris-HCl, pH 7, 250 mM NaCl, 2 mM DTT, the protein complexes on the beads were eluted by 10 mM glutathione. All samples were treated with SDS-loading buffer, boiled at 95 ℃ for 5 minutes and resolved by SDS-PAGE. Proteins were analyzed as indicated by Coomassie staining. Bio-Layer Interferometry real-time binding assay of DDA1-DDB1 were performed with a Pall FortéBio Octet system according to the manufacture instruction. Briefly, GST-tagged DDA1 proteins were loaded on anti-GST biosensors, non-tagged DDB1 protein samples were titrated in serial dilution (10, 3.3, 1.1, 0.37, 0.124, 0.042 μM). All steps were performed at 25 ℃ in a buffer containing 40 mM Tris-HCl pH 7, 100 mM NaCl, 2 mM DTT with an agitation speed of 1000 rpm. The

sensorgrams were referenced against the buffer reference signal and the empty sensors by the Data Analysis software (ForteBio). Data of $R_{equilibrium}$ were analyzed and fitted by Prism 6.

Ubiquitination of DDA1 in a reconstituted cell-free system

GST-tagged DDA1 proteins were incubated at 37 ℃ for 1 h (in a volume of 12.5 μL) in the presence of 5 μg of either Ub (WT) or Lysine-Less Ub (LL-Ub, all internal Lys residues were substituted with Arg), E1 (0.25 μg), ATP-$MgCl_2$ (0.5 mM), E2s (mixture of UbcH5 and Ube2g, 0.3 μg), purified CUL4A-DDB1 (0.4 μg). Ub, LL-Ub, and enzymes were expressed and purified as described above and previously[1,6]. All reactions were terminated by the addition of 5-fold concentrated sample buffer. Samples were resolved by SDS-PAGE and visualized via Western blot using anti-GST antibody (Sigma).

Supplementary Table S1. Data collection and refinement statistics

	DDA1-DDB1
**Data collection**	
Space group	$P2_1$
Cell dimensions	
a, *b*, *c* (Å)	63.2 219.2 89.3
α, β, γ (°)	90 90 90
Resolution (Å)	47.8 - 3.093 (3.2-3.1)
R_{sym}	0.136 (0.586)
$I / \sigma I$	10.29 (1.62)
Completeness (%)	89 (49)
Redundancy	2.7 (1.6)
**Refinement**	
Resolution (Å)	3.1
No. reflections	39217
R_{work} / R_{free} (%)	24.8/30.5
No. atoms	17588
Protein	17588
Ligand/ion	0
Water	0
B-factors	
Protein	51.87
Ligand/ion	N/A
Water	N/A
R.m.s. deviations	
Bond lengths (Å)	0.007
Bond angles (°)	1.9
Ramachandran favored (%)	85
Ramachandran allowed (%)	15
Ramachandran outliers (%)	0.36
PDB ID	6DSZ

References

1 Angers, S. *et al.* Molecular architecture and assembly of the DDB1-CUL4A ubiquitin ligase machinery. *Nature* **443**, 590-593, doi:10.1038/nature05175 (2006).

2 Otwinowski, Z. & Minor, W. Processing of X-ray diffraction data collected in oscillation mode. *Methods Enzymol* **276**, 307-326 (1997).

3 Li, T., Chen, X., Garbutt, K. C., Zhou, P. & Zheng, N. Structure of DDB1 in complex with a paramyxovirus V protein: viral hijack of a propeller cluster in ubiquitin ligase. *Cell* **124**, 105-117, doi:10.1016/j.cell.2005.10.033 (2006).

4 Adams, P. D. *et al.* PHENIX: a comprehensive Python-based system for macromolecular structure solution. *Acta Crystallogr D Biol Crystallogr* **66**, 213-221, doi:10.1107/S0907444909052925 (2010).

5 Emsley, P., Lohkamp, B., Scott, W. G. & Cowtan, K. Features and development of Coot. *Acta Crystallogr D Biol Crystallogr* **66**, 486-501, doi:10.1107/S0907444910007493 (2010).

6 Shabek, N. *et al.* The size of the proteasomal substrate determines whether its degradation will be mediated by mono- or polyubiquitylation. *Mol Cell* **48**, 87-97, doi:10.1016/j.molcel.2012.07.011 (2012).
